# Complete Genome Assembly of *Pantoea stewartii* subsp. *stewartii* DC283, a Corn Pathogen

**DOI:** 10.1128/genomeA.00435-17

**Published:** 2017-06-01

**Authors:** Duy An Duong, Ann M. Stevens, Roderick V. Jensen

**Affiliations:** Department of Biological Sciences, Virginia Tech, Blacksburg, Virginia, USA

## Abstract

The phytopathogen *Pantoea stewartii* subsp. *stewartii* DC283 causes Stewart’s wilt disease in corn after transmission from the corn flea beetle insect vector. Here, we report that the complete annotated genome of *P. stewartii* DC283 has been fully assembled into one circular chromosome, 10 circular plasmids, and one linear phage.

## GENOME ANNOUNCEMENT

*Pantoea stewartii* subsp. *stewartii* is a Gram-negative gammaproteobacterium native to North America which causes Stewart’s wilt disease in corn, resulting in economic loss and quarantine of exports. Transmission to plants occurs primarily through seeds or inoculation from the corn flea beetle (1, 2). *P. stewartii* DC283 is the wild-type reference strain used to study pathogenesis (3–5). It is a nalidixic acid-resistant mutant of the original 1976 isolate, SS104, from *Zea mays* (6, 7). In 2012, a draft assembly of the genome with 65 contigs (NCBI GenBank accession no. AHIE00000000.1) was released. However, the completion of this genome has been complicated by large numbers of repetitive transposon sequences often spanning 1,500 bp. Using Illumina mate-pair sequencing with 3,500-bp insert size, this study reports the complete genome assembly of *P. stewartii* DC283.

Genomic DNA was extracted from an overnight culture of *P. stewartii* DC283 grown in Luria-Bertani medium using a Qiagen DNeasy blood and tissue kit, as per the manufacturer’s recommendations for Gram-negative bacteria. On-column RNase treatment was applied before DNA elution to yield high-quality DNA for Nextera mate-pair preparation and Illumina MiSeq sequencing using 250-bp mate-pair reads with 3,500-bp inserts. The mate-pair reads were first aligned to the 65 contigs of the reference genome using the Geneious version 9.1.2 software package (Biomatters Ltd.) “Map to Reference” function. Unmapped mate-pair reads were then *de novo* assembled to identify missing sequences and rearrangements of the reference contigs. Finally, the end sequences of all of the remaining contigs were extended ~3,500 bp by *de novo* assembling mate-pair reads, where one mate maps to the last 3,500 bp of the contig. This procedure was sufficient to link most of the reference contigs, leading to the complete assembly of the 10 circular plasmids and of the main chromosome into 3 large segments. The chromosome segments were connected into a closed circular sequence using long-range PCR (Qiagen LongRange PCR kit). In addition, a linear phage was identified with similarity to the N15 phage-plasmid in *Escherichia coli* (8). This resulted in ~200× coverage of the genomic sequence and between ~200× and ~3,000× coverage for the plasmids.

Automatic annotation for the *P. stewartii* DC283 genome was performed using the NCBI Prokaryotic Genome Annotation Pipeline (9, 10). The whole genome consists of 5,314,092 bp (53.8% G+C content), with 5,625 coding sequences, 21 rRNAs, and 73 tRNAs. The chromosome is 4,528,215 bp, the plasmids range in size from 4,277 to 304,641 bp, and the linear phage is 47,186 bp. More than 460 sequences encoding repetitive transposases were found in the complete genome. Interestingly, the two type III secretion systems that play important roles in the colonization of insect and plant hosts (11) were found to be located on two separate megaplasmids. In addition, a 66-kb region was newly assembled in this genome compared to the reference genome.

### Accession number(s).

The annotated genome assembly of *P. stewartii* DC283 is available in GenBank under accession numbers CP017581 to CP017592.
